# Supplementary material for: Bridging Genomics and Clinical Medicine: RSVrecon Enhances RSV Surveillance With Automated Genotyping and Clinically Important Mutation Reporting
Source: Influenza Other Respir Viruses. 2026 Jan 4;20(1):e70203. doi: 10.1111/irv.70203 (PMC12765627; doi:10.1111/irv.70203)
Supplement: Supplementary file 2 — Figure S1: Phylogenetic tree including assembled sequences and reference. Sequences generated from different pipelines are indicated by colors. A: Phylogenetic tree of tested samples of subtype A. B: Phylogenetic tree of tested samples of subtype B. [file IRV-20-e70203-s001.pdf]

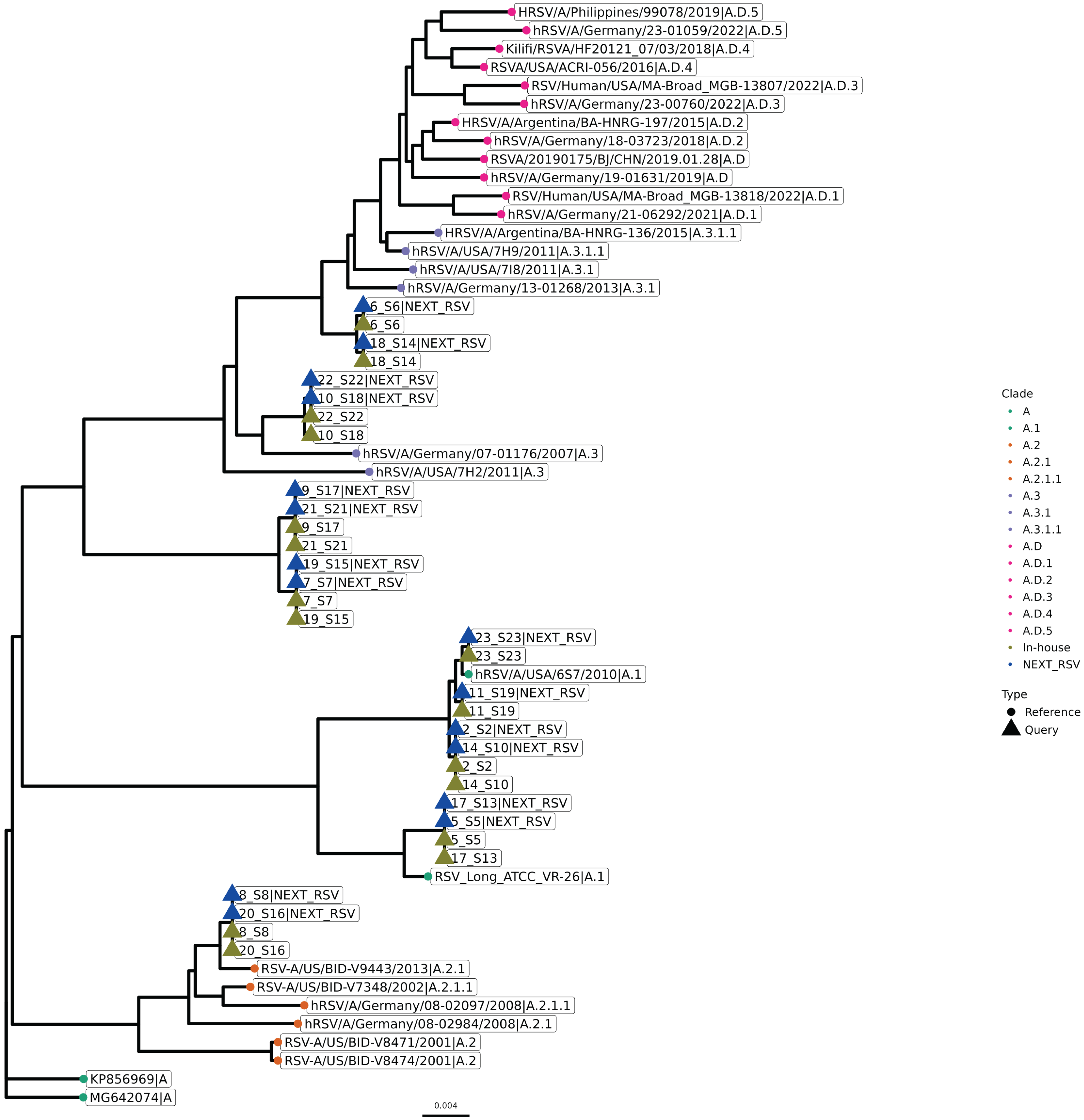

Figure S1. Phylogenetic tree including assembled sequences and reference. Sequences generated from different pipelines are indicated by colors. A: Phylogenetic tree of tested samples of subtype A. B: Phylogenetic tree of tested samples of subtype B.
