# Supplementary material for: Bridging Genomics and Clinical Medicine: RSVrecon Enhances RSV Surveillance With Automated Genotyping and Clinically Important Mutation Reporting
Source: Influenza Other Respir Viruses. 2026 Jan 4;20(1):e70203. doi: 10.1111/irv.70203 (PMC12765627; doi:10.1111/irv.70203)
Supplement: Supplementary file 3 — Figure S2: Accessible and user‐friendly outputs of RSVrecon. The pipeline generates two levels of comprehensive reports: (1) a batch‐level summary displaying QC metrics, mapping quality, subtypes, genotypes, gene coverage profiles, and phylogenetic relationships across all samples; and (2) detailed sample‐specific reports featuring assembly quality metrics, genotype characterization, F protein mutations, best‐matching reference sequences, whole‐genome phylogenetic placement, coverage statistics, and SNP annotations. [file IRV-20-e70203-s002.pdf]

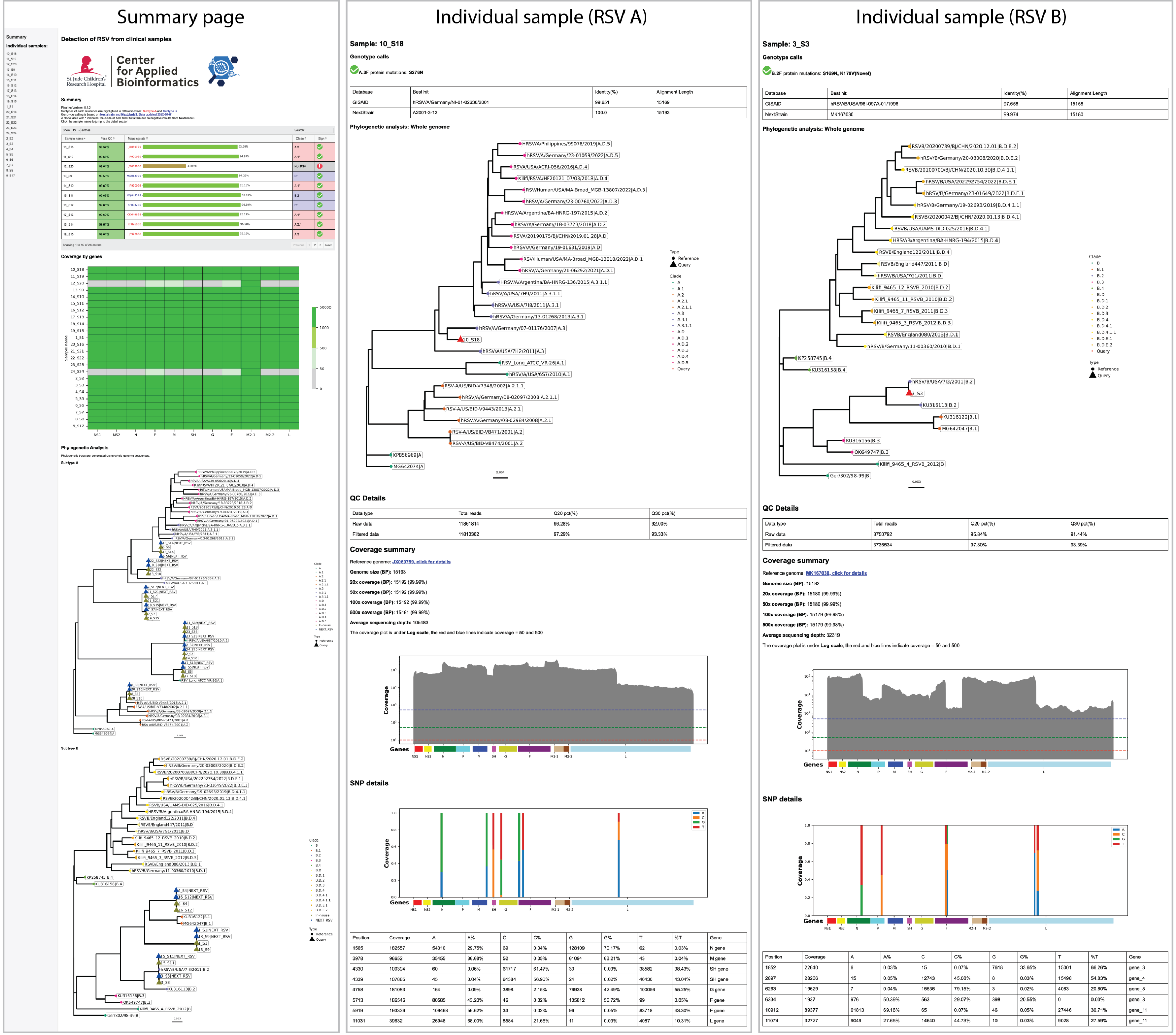

Figure S2. Accessible and User-Friendly Outputs of RSVrecon. The pipeline generates two levels of comprehensive reports: (1) a batch-level summary displaying QC metrics, mapping quality, subtypes, genotypes, gene coverage profiles, and phylogenetic relationships across all samples; and (2) detailed sample-specific reports featuring assembly quality metrics, genotype characterization, F protein mutations, best-matching reference sequences, whole-genome phylogenetic placement, coverage statistics, and SNP annotations.
